# Allele-Specific Expression of CD4^+^ T Cells in Response to Marek’s Disease Virus Infection

**DOI:** 10.3390/genes10090718

**Published:** 2019-09-17

**Authors:** Hao Bai, Yanghua He, Yi Ding, José A. Carrillo, Ramesh K. Selvaraj, Huanmin Zhang, Jilan Chen, Jiuzhou Song

**Affiliations:** 1 Joint International Research Laboratory of Agriculture and Agri-Product Safety, the Ministry of Education of China, Institutes of Agricultural Science and Technology Development, Yangzhou University, Yangzhou 225009, China; 2 Department of Animal & Avian Sciences, University of Maryland, College Park, MD 20742, USA; 3 Key Laboratory of Animal (Poultry) Genetics Breeding and Reproduction, Ministry of Agriculture, Institute of Animal Science, Chinese Academy of Agricultural Sciences, Beijing 100193, China; 4 USDA, Agricultural Research Service, Avian Disease and Oncology Laboratory, East Lansing, MI 48823, USA; 5 Department of Animal Sciences, The Ohio State University, Wooster, OH 44691, USA; 6 Department of Human Nutrition, Food and Animal Sciences, University of Hawaii at Manoa, HI 96822, USA

**Keywords:** Marek’s disease virus, CD4^+^ T cells, allele-specific expression, differential expression, genetic resistance

## Abstract

Marek’s disease (MD) is a T cell lymphoma disease induced by Marek’s disease virus (MDV), a highly oncogenic α herpesvirus primarily affecting chickens. MD is a chronic infectious disease that threatens the poultry industry. However, the mechanisms of genetic resistance for MD are complex and not completely understood. In this study, to identify high-confidence candidate genes of MD genetic resistance, high throughput sequencing (RNA-seq) was used to obtain transcriptomic data of CD4^+^ T cells isolated from MDV-infected and non-infected groups of two reciprocal crosses of individuals mating by two highly inbred chicken lines (6_3_ MD-resistant and 7_2_ MD-susceptible). After RNA-seq analysis with two biological replicates in each group, we identified 61 and 123 single nucleotide polymorphisms (SNPs) (false discovery rate (FDR) < 0.05) annotated in 39 and 132 genes in intercrosses 6_3_ × 7_2_ and 7_2_ × 6_3_, respectively, which exhibited allele-specific expression (ASE) in response to MDV infection. Similarly, we identified 62 and 79 SNPs annotated in 66 and 96 genes in infected and non-infected groups, respectively. We identified 534 and 1543 differentially expressed genes (DEGs) (FDR < 0.05) related to MDV infection in intercrosses 6_3_ × 7_2_ and 7_2_ × 6_3_, respectively. We also identified 328 and 20 DEGs in infected and non-infected groups, respectively. The qRT-PCR using seven DEGs further verified our results of RNA-seq analysis. The qRT-PCR of 11 important ASE genes was performed for gene functional validation in CD4^+^ T cells and tumors. Combining the analyses, six genes (*MCL1*, *SLC43A2*, *PDE3B*, *ADAM33*, *BLB1*, and *DMB2*), especially *MCL1*, were highlighted as the candidate genes with the potential to be involved in MDV infection. Gene-set enrichment analysis revealed that many ASE genes are linked to T cell activation, T cell receptor (TCR), B cell receptor (BCR), ERK/MAPK, and PI3K/AKT-mTOR signaling pathways, which play potentially important roles in MDV infection. Our approach underlines the importance of comprehensive functional studies for gaining valuable biological insight into the genetic factors behind MD and other complex traits, and our findings provide additional insights into the mechanisms of MD and disease resistance breeding in poultry.

## 1. Introduction

Marek’s disease (MD) is a T cell lymphoma and a strictly cell-associated disease induced by the highly oncogenic α-herpesvirus II disease virus [1], which has a complex life with four main phases [2,3]: an early cytolytic phase at 2–7 days post infection (dpi), a latent phase around 7–10 dpi, a late cytolytic phase with the presence of tumors that is triggered between 14 and 21 dpi, and a final proliferation phase after 28 dpi. During the first cytolytic phase, Marek’s disease virus (MDV) first uses B cells as targets for its replication before targeting activated CD4^+^ T cells to enable a persistent latent infection [4,5,6]. MDV then uses T cells as the main target during the later phase, especially CD4^+^ T cells [7] during the cytolytic phase with tumors at 21 dpi, which were used in this study. CD4 is a co-receptor that facilitates T cell receptor (TCR) communication with an antigen-presenting cell. MD is a commercially important neoplastic disease in chickens and also the main chronic infectious disease threatening the poultry industry. Enhancing genetic resistance to MD in poultry is an important long-term goal in controlling MD. To optimally implement this control strategy through marker assisted selection (MAS) and to understand the etiology and mechanisms of MD, it is necessary to identify more specific alleles and genes with respect to MD latency, although alleles at the well-studied major histocompatibility complex (MHC) locus are already known to be involved in genetic resistance to MD.

The comprehensive identification of genes underlying phenotypic variation of complex traits, especially disease resistance, remains one of the greatest challenges in biology, despite having genome sequences and powerful tools. Screening for allele-specific expression (ASE) [8,9,10,11] is an effective approach to identifying regulatory variation responsible for differences in transcript abundance in genes. ASE refers to unequal expression of multiple alleles of a gene, and the extreme case of ASE is monoallelic expression, where only one of the alleles is expressed while the other is completely inactive. The genes with single nucleotide polymorphisms (SNPs) exhibiting ASE will provide a strong foundation for investigating the genetic mechanisms of complex traits, such as MDV infection. Cheng et al. [12] found that SNPs in ASE genes account for more than 83% of the additive genetic variation of genetic resistance to MD, demonstrating that most of the genes exhibiting ASE are strong candidates for studying MD resistance. MacEachern et al. [13] suggested that ASE can be used to identify genes with cis-regulatory elements that respond to MDV infection. Perumbakkam et al. [14] considered that the TLR and JAK/STAT signaling pathways may be responding to MDV infection through the genes exhibiting ASE.

In the present study, transcriptomic data in CD4^+^ T cells isolated from MDV-infected and non-infected groups of two reciprocal crosses were analyzed by high throughput sequencing (RNA-seq) to identify candidate genes of MD genetic resistance. Two F1 reciprocal crosses were used to compare the differences, not only between infected and non-infected individuals, but also between two reciprocal crosses. Key SNPs, genes, and pathways related to MD resistance or MD susceptibility were identified by ASE and differential expression (DE) analyses. We used both CD4^+^ T cells and tumors for the validation of genes exhibiting ASE.

## 2. Materials and Methods

### 2.1. Ethics Statement

All of the chickens were kept in a pathogen-free facility at the Avian Disease and Oncology Laboratory (ADOL, East Lansing, MI, USA). All animals were approved by the United States Department of Agriculture (USDA), Agricultural Research Service, and ADOL Animal Care and Use Committee. Their guidelines (revised April 2005, Approval Number: 6040-31320-009-00-D) were followed, along with the Guide for the Care and Use of Laboratory Animals published by Institute for Laboratory Animal Research (ILAR Guide) in 1996 (http://www.nap.edu/openbook.php?record_id=5140) and University of Maryland (R-08-62). All efforts were made to minimize discomfort and suffering.

### 2.2. Chickens, Samples, and Experimental Design

Two reciprocal crosses were obtained—the F1 progeny from intermating 6_3_ and 7_2_ lines (USDA-ARS ADOL, East Lansing, MI, USA) [15], two highly inbred chicken lines that are MD-resistant and MD-susceptible, respectively. For each reciprocal cross, the chickens were divided into two groups, with two birds infected with MDV and two non-infected controls. A very virulent plus (vv+) strain of MDV-648A passage 50 [16] was injected intra-abdominally at day 5 after hatching with a viral dosage of 500 plaque-forming units (PFU) per chick. The viral challenge experiment was conducted in the BSL-2 facility at ADOL. At 21 dpi, CD4^+^ T cells were isolated from blood using a CD4^+^ T cell isolation kit (Miltenyi Biotec, Auburn, CA, USA) with anti-biotin microbeads (Miltenyi Biotec, Auburn, CA, USA) and mouse anti-chicken CD4 antibody (SouthernBiotech, Birmingham, AL, USA). The number and purities of enriched cells were determined by flow cytometry using the MACSQuant^®^ analyzer (Miltenyi Biotec, Auburn, CA, USA) and stored in RNAlater solution (Qiagen, Valencia, CA, USA) immediately at −80 °C until RNA extraction.

### 2.3. Library Construction and Sequencing

Total RNA was isolated from CD4^+^ T cells using TRIzol (Invitrogen, Carlsbad, CA, USA) and examined using the RNeasy Mini Kit (Qiagen, Valencia, CA, USA) according to the manufacturer’s instructions. RNA concentration was assessed using a Nanodrop ND-1000 spectrophotometer (Thermo Scientific, Madison, WI, USA) and RNA quality was determined using the 2100 Bioanalyzer (Agilent, Pal Alto, CA, USA). Only samples with RIN scores over 7 could be submitted for sequencing. Then, mRNA was used to synthesize the first- and second-strand cDNA using SuperScriptTM III reverse transcriptase (Invitrogen, Carlsbad, CA, USA) and oligo (dT) 12–18 primers (Invitrogen, Carlsbad, CA, USA). After purification, the double-strand cDNA (dscDNA) was fragmented into ~300 base pairs (bp). The library was subsequently constructed as follows. End repair of the fragmented dscDNA was performed and then 3’ poly-A was added to the end-repaired dscDNA using DNA polymerase I, large (Klenow) fragment. A pair of Solexa adaptors was ligated to the repaired ends using T4 ligase, then 200–400 bp of fragments were selected using the Invitrogen^®^ 2% E-Gel (Carlsbad, CA, USA). Specific dscDNA fragments were amplified by PCR and the libraries were then quantified and pooled. Finally, clusters were generated and sequencing was analyzed using Illumina Hiseq 2000 (single end, 50 base read length; San Diego, CA, USA). The sequencing data were submitted to the Sequence Read Archive (SRA) of National Center for Biotechnology Information (NCBI), and are accessible through the accession number PRJNA488865.

### 2.4. Mapping and Assembling

Chicken genome assembly (galGal4) was downloaded from UCSC Genome Browser website (http://hgdownload.soe.ucsc.edu/goldenPath/galGal4/bigZips/) [17]. To minimize the mapping errors, quality control was performed by FastQC [18] and low quality reads were removed with the help of the FastX Toolkit [19] and Trimmomatic [20] with default parameters. The resulting FastQ files of mapping reads of each sample were individually aligned with the reference genome using Bowtie 2 (version 2.2.7) [21,22], with mainly default parameters. SAMtools (version 1.3) [23] was then used to convert the alignment results (SAM format) to BAM format for calling SNPs (for ASE analysis) and counting reads number (for DE analysis).

### 2.5. SNP Calling, ASE Estimation, and Functional Annotation of SNPs

SNP calling was performed for each chromosome on all samples simultaneously, using SAMtools and BCFtools (version 1.3) [24] with basic command lines. The SNPs were then filtered using the BCFtools to exclude the following variants: (1) SNPs with a mapping quality covering reads < 20; and (2) SNPs with a total read depth < 4 or > 100. Read counts per allele were calculated by the Genome Analysis Toolkit (GATK, version 3.5) [25] ASEReadCounter with default parameters. The read counts of each allele were used for further ASE SNP detection. All of the indels were subsequently removed and the variant calling format (VCF) files derived from non-infected and infected birds were merged using VCFtools (version 0.1.14) [26]. An in-house R script was used for allelic imbalance determined by a chi-square test with two variables: (1) the read counts (allele A (reference) and B (alternative)) of each SNP and (2) two different groups (four comparison groups were divided into 6_3_ × 7_2_ infected (I) vs 6_3_ × 7_2_ non-infected (N), 7_2_ × 6_3_ I vs 7_2_ × 6_3_ N, 6_3_ × 7_2_ I vs 7_2_ × 6_3_ I, and 6_3_ × 7_2_ N vs 7_2_ × 6_3_ N). SNPs with a 0.05 level of significance (false discovery rate (FDR) < 0.05) were chosen for further investigation. Variant Effect Predictor (VEP) [27] based on the Ensembl website (http://useast.ensembl.org/Gallus_gallus/Tools/VEP) was used to functionally annotate the putative SNPs to the genes within 5000 bp. Each SNP was classified based on its position in the reference chicken genome as exonic, intronic, intergenic, 5’ untranslated region (UTR), 3’ UTR, splicing site, and upstream or downstream.

### 2.6. Differential Expression (DE) Analysis

The alignments obtained above were counted by HTSeq count based on python [28] and analyzed using edgeR [29], a bioconductor package for DE analysis within R. The resulting data of HTseq from all of the individual samples were converted and merged to text files imported into R and then analyzed. A portion of the reads (10.4 and 13.8 million reads on average for intercross 6_3_ × 7_2_ and 7_2_ × 6_3_, and 11.8 and 12.4 million reads on average in infected and non-infected groups, respectively) were assigned as “no feature”.

### 2.7. Experimental Validation

To evaluate the reliability of our data analysis, we randomly selected seven genes for quantitative real-time PCR (qRT-PCR) validation in CD4^+^ T cells. Simultaneously, 11 overlapped genes between ASE and DE analysis were selected for gene functional validation in both CD4^+^ T cells and tumors. The protocols followed for mRNA extraction and dsDNA synthesis were the same as those mentioned above. Gene-specific primers were designed to span over exons from the chicken genome reference Unigene sequences available in GenBank for conventional PCR amplification using Primer3 (http://fokker.wi.mit.edu/primer3/input.htm) and confirmed by Oligo 6.0 (Tables S1 and S2). The housekeeping gene *GAPDH* and *β-actin* were used as the endogenous control. The qRT-PCR using SYBR Green PCR Kit was performed in triplicate based on iCycler iQ PCR System (Bio-Rad, Hercules, CA, USA). The qRT-PCR reaction program was run as follows: pre-incubation (95 °C for 10 min), 40 cycles of amplification (95 °C for 10 s, 60 °C for 10 s, and 72 °C for 10 s), melting curves using a heat ramp, and cool down. Cycle threshold values (Ct values) were obtained from iCycler iQ PCR software (Bio-Rad, Hercules, CA, USA). The expressions of genes were normalized against *GAPDH* or β-actin cDNA in the corresponding samples. The relative fold enrichment of each treatment group was calculated by comparing the enrichment value to *GAPDH* or β-actin.

### 2.8. Gene Ontology (GO) and Pathway Enrichment Analyses Using DAVID and IPA

Two methods were used for GO and pathway enrichment analysis here. The ASE significant genes (FDR < 0.05) list was firstly submitted to DAVID (version 6.7) (https://david-d.ncifcrf.gov/) [30]. The analysis classification stringency was set to the highest level with suitable controls. The resulting clustering was then limited to an enrichment score of >1.0 and FDR for multiple testing was performed following the Benjamin and Hochberg method. Kyoto Encyclopedia of Genes and Genomes (KEGG) pathway analysis was also performed using DAVID. Simultaneously, gene functions were annotated using the Ingenuity Pathway Analysis (IPA) tool (http://www.ingenuity.com/products/ipa). The program inquires the IPA knowledge database for information and direct relationships of genes and endogenous chemicals. This creates algorithmically generated networks and data clustered into biological functions and diseases that are overrepresented in the scrutinized data. The program determines the high representation of signaling and biological pathways. The method employs the Fisher’s exact test, determining the proportion of genes mapped to a function or pathway in the sample and then compares it to the ratio in the reference set. The result included functional networks, disease and disorders, molecular and cellular functions, physiological system development and function, top canonical pathways, upstream regulators, and top toxicity lists.

## 3. Results

### 3.1. ASE SNP Discovery and ASE Genes in Response to MDV Infection

**Figure 1:**
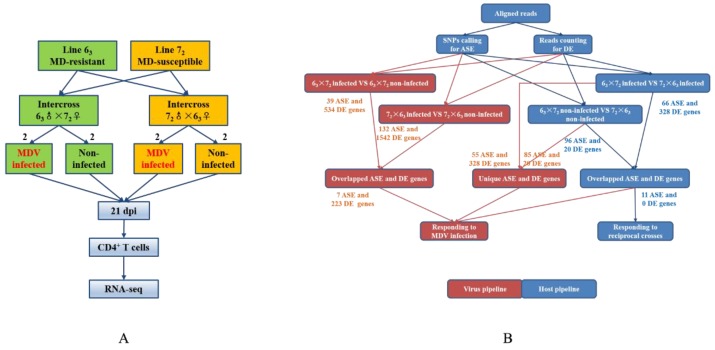
Experimental and data analyses designs. (**A**) Experimental design for identifying allele-specific expression (ASE) and differentially expressed (DE) genes based on high throughput sequencing (RNA-seq) using reciprocal crosses. (**B**) The pipeline of ASE and DE gene identification. The number on an arrow indicates the remaining genes after the previous step.

In the present study, we sequenced RNA samples from two infected and two non-infected birds for each reciprocal cross (Figure 1). The average number of raw reads was approximately 22.15, 28.92, 23.37, and 27.69 million for intercross 6_3_ × 7_2_, 7_2_ × 6_3_, infected, and non-infected datasets, respectively (Table 1). After quality control (QC) procedures, all of the raw data were sufficient, with no further trimming processes required. The mapping levels of the samples were adequate and similar, ranging from 73.13% to 79.61% for all individuals. These high quality alignments were appropriate for subsequent analysis, with few false positives.

All of the SNPs were called within alignments using SAMtools and BCFtools, and read counts per allele were calculated with the help of GATK ASEReadCounter (Broad Institute, Cambridge, MA, USA). The average number of SNPs finally called was in the approximate range of 500,000 to 710,000. With the criteria described above, around 150,000–260,000 SNPs were left after filtering, and about one-third of heterozygous SNPs were left (Table 2). All of the VCF files were then merged and divided into four comparison groups (infection in contrast to non-infection in 6_3_ × 7_2_ and 7_2_ × 6_3_ intercross; infection comparison and non-infection comparison groups between two intercrosses) using VCFtools and consensus SNPs (the missing SNPs that were not present in all of the individuals involved in one comparison group were removed and the overlapped SNPs were retained) were ultimately selected for ASE SNP identification (Table 3).

**Figure 2:**
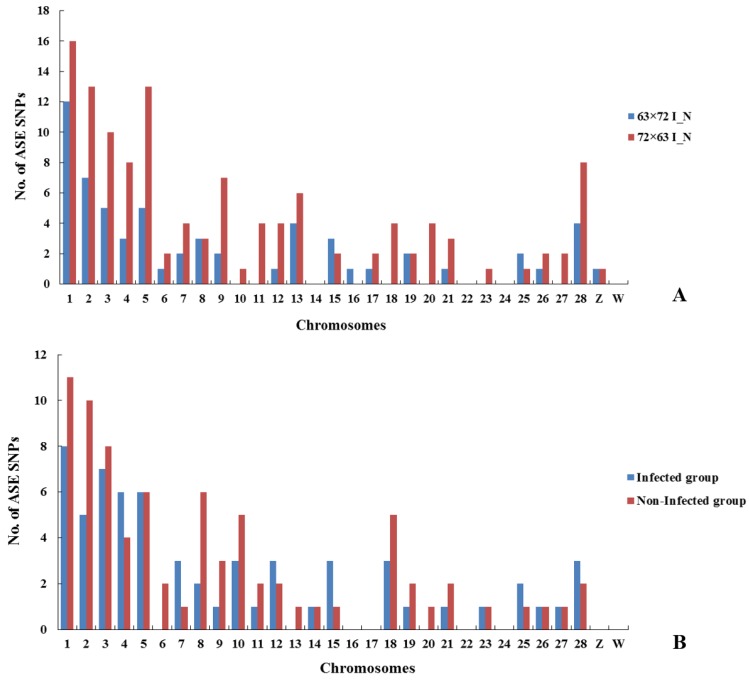
Distribution of ASE SNPs in chicken chromosomes. (**A**) Distribution of ASE SNPs in intercrosses 6_3_ × 7_2_ and 7_2_ × 6_3_, respectively, in response to MDV infection. (**B**) Distribution of ASE SNPs between two reciprocal crosses in infected and non-infected groups.

An R script was used for each SNP determined by a chi-square test examining each allele (read counts of alleles A and B) and two different statuses in birds—infected and non-infected. A total number of 61 and 123 SNPs exhibiting ASE in response to MDV infection were identified in intercrosses 6_3_ × 7_2_ and 7_2_ × 6_3_, respectively (FDR < 0.05, Table 3). The significant SNPs for both reciprocal crosses were distributed on each chromosome of the chicken genome (Figure 2A). To locate these SNPs on the chicken genome with respect to genes and classify them based on function, VEP was then used to functionally annotate the putative SNPs to the genes within 5000 bp. In intercross 6_3_ × 7_2_, 61 significant SNPs were located in 39 genes; the majority of SNPs were classified as exonic, intronic, and intergenic. Similarly, in intercross 7_2_ × 6_3_, 123 SNPs were located in 132 genes; the majority of SNPs were classified as intronic, downstream, and exonic (Table 4). For example, two alleles with T-A and A-G on chromosome 25 are located on the intron of *MCL1*. Three alleles with C-A, A-T, and T-C on chromosome 5 are located on the intron of *PDE3B*. Seven genes were matched between the two reciprocal crosses, including *MCL1*, *PTBP1*, *SLC43A2*, *ST6GAL1*, *PDE3B*, *RUNX1*, and *ADAM33*. The detailed lists of ASE SNPs and genes responding to MDV infection are provided in Table S3.

### 3.2. ASE SNP Discovery and ASE Genes between Two Chicken Reciprocal Crosses

Similarly, a chi-square test was used to examine each allele and two different statuses in infected birds of intercross 6_3_ × 7_2_ compared with those of 7_2_ × 6_3_ and in non-infected birds. A total of 62 and 79 SNPs exhibiting ASE were identified in infected and non-infected birds, respectively (FDR < 0.05, Table 3). The significant SNPs were distributed on each chromosome of the chicken genome (Figure 2B). A total of 62 significant SNPs were located in 66 genes and 79 SNPs in 96 genes in infected and non-infected groups, respectively (Table S3). The classification of ASE SNPs is detailed in Table 5. Therefore, 96 ASE genes were identified before MDV infection and 66 were identified after infection, with 11 overlapped genes in total (*MCL1*, *VAV3*, *PTPRC*, *RAB8B*, *ATP13A2*, *RAB43*, *CHD2*, *CD44*, *NRK*, *RHOH*, and *ENSGALG00000026782* (novel gene)), including *MCL1*. Interestingly, the same alleles located on the intron of *MCL1* were also identified. The common genes were both identified before and after MDV infection, so they may not only be associated with MDV but also be related to reciprocal crosses in both the virus- and host-related genes. We identified 85 unique genes before infection and 55 unique genes after infection, which should be in response to MDV infection with different statuses before and after infection.

### 3.3. Comparison and Analysis of Differentially Expressed Genes (DEGs)

**Figure 3:**
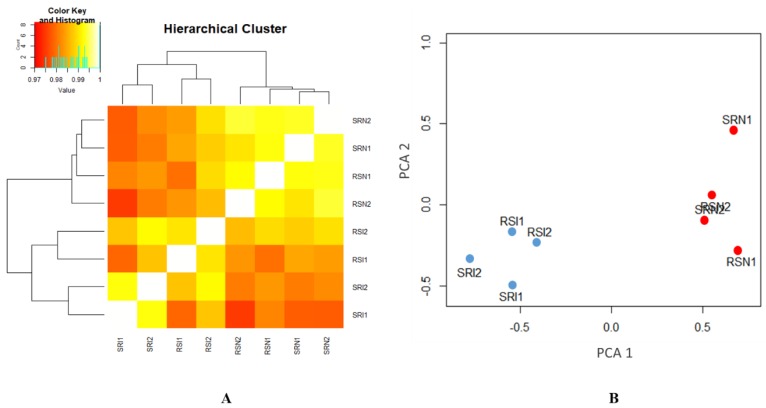
Cluster map of all of the samples. (**A**) Heatmap and hierarchical clustering tree based on the Reynolds’ distance between individuals. RS and SR represent intercrosses 6_3_ × 7_2_ and 7_2_ × 6_3_, respectively. I and N respect infected and non-infected groups, respectively. Two biological replicates are clustered together. (**B**) Principle component analysis (PCA) plots based on the first two principal components. The blue and red dots represent infected and non-infected birds, respectively. The infected and non-infected birds are clustered into two groups.

**Figure 4:**
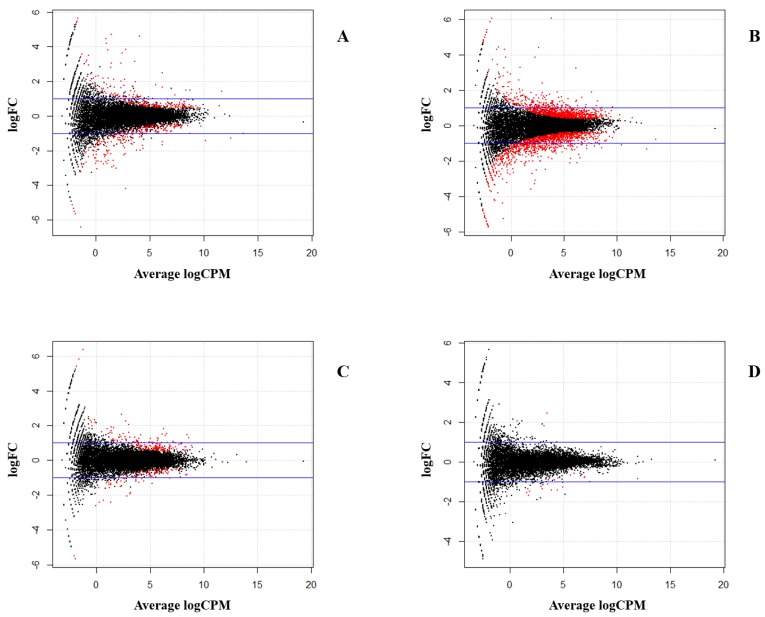
The differentially expressed genes. (**A**) Infected birds in contrast to non-infected birds in intercross 6_3_ × 7_2_. (**B**) Infected birds in contrast to non-infected birds in intercross 7_2_ × 6_3_. Comparison of two reciprocal crosses in (**C**) infected groups and (**D**) non-infected groups. MA plot shows the significant differentially expressed genes in red (y-axis depicts the log_2_ fold change (FC) of gene expression; x-axis represents the average log reads count per million (CPM) for each gene; the blue lines denote |log_2_ Fold Change| = 1).

The same aligned datasets were used for the DE analysis. DE was estimated by obtaining the count data from each biological sample using the Htseq script [28]. Biological replicates were closed to each other (Figure 3) to verify the reliability of our datasets. A total of 17,108 chicken genes from galGal4 and edgeR, based on R package, were then used to estimate DE. We also divided the samples into four comparison groups the same as for ASE analysis. A total of 534 and 1543 DEGs related to MDV infection were identified in intercrosses 6_3_ × 7_2_ and 7_2_ × 6_3_, respectively (FDR < 0.05; Table 6, Figure 4A,B). For intercross 6_3_ × 7_2_, the expression of 382 (71.5%) DEGs were higher and 152 (28.5%) were lower after infection. For intercross 7_2_ × 6_3_, 854 (55.3%) and 689 (44.7%) DEGs were up- and down-regulated after infection, respectively. Similarly, for 328 and 20 DEGs, 279 up-regulated and 49 down-regulated genes were identified in infected groups, and 5 up-regulated and 15 down-regulated genes were identified in non-infected groups (FDR < 0.05; Table 6, Figure 4C,D), respectively. The detailed lists of DEGs are provided in Table S4. Compared with the ASE genes, 3 and 24 overlapped genes responding to MDV infection were identified in intercrosses 6_3_ × 7_2_ and 7_2_ × 6_3_, respectively. Similarly, 10 and 0 overlapped genes were identified in infected groups and non-infected groups, respectively (Table 7). These common genes, including *MCL1*, *SLC43A2*, *PDE3B*, and *ADAM33*, which were mentioned above, could be selected as candidate MD-resistant or MD-susceptible genes. The detailed information of these genes is provided in Table S5.

### 3.4. RNA-Seq Results Verification and Gene Functional Validation by qRT-PCR

**Figure 5:**
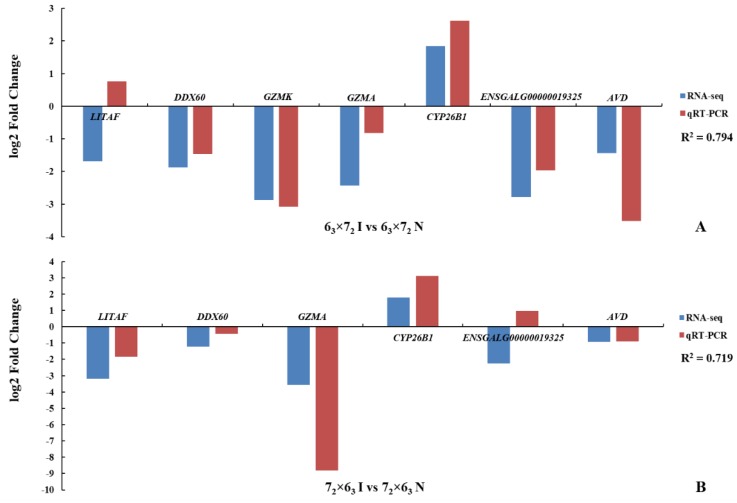
The qRT-PCR of seven genes for validating the RNA-seq results in intercrosses (**A**) 6_3_ × 7_2_ and (**B**) 7_2_ × 6_3_. The y-axis represents the log_2_ FC of gene expression; the x-axis shows the Ensembl names of genes employed for validation. Blue and red bars depict the RNA-seq and qRT-PCR results, respectively. *R^2^* refers to the coefficient of determination.

To verify the RNA-seq analysis, seven genes (*GZMA*, *GZMK*, *LITAF*, *DDX60*, *AVD*, *CYP26B1*, and *ENSGALG00000019325* (novel gene)) were examined by qRT-PCR using the RNA of CD4^+^ T cells derived from infected and non-infected birds. The results (Figure 5) showed good agreement with the DE analysis pattern, indicating the reliability of the latter.

**Figure 6:**
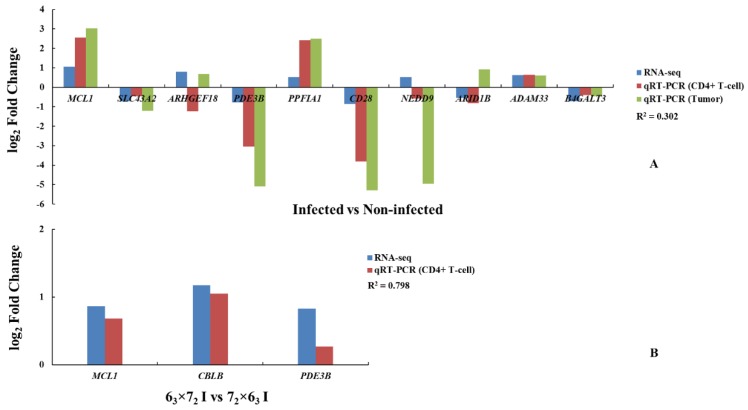
Gene functional validation in CD4^+^ T cells and tumors using qRT-PCR: (**A**) Infected in contrast to non-infected; (**B**) infected comparison groups between two reciprocal crosses. The y-axis represents the log_2_ FC of gene expression; the x-axis shows the genes employed for validation. Blue, red, and green bars depict the RNA-Seq and qRT-PCR of genes in CD4^+^ T cell and tumor results, respectively. *R^2^* refers to the coefficient of determination.

As described above, the overlapped genes between ASE and DE analyses may be much more important. Thus, we selected 11 overlapped genes based on their existing gene functions and the position of their ASE SNPs for the functional validation in both CD4^+^ T cells and tumors using qRT-PCR to confirm whether they are critical genes in the response to MDV infection. The results (Figure 6) revealed that some of these genes, including *MCL1*, *SLC43A2*, *PDE3B*, *ADAM33*, *PPFIA1*, *CD28*, *B4GALT3*, and *CBLB*, showed significant differences (|fold change (FC)| > 1.2 or |log_2_ FC| > 0.27) in both CD4^+^ T cells and tumors of the comparison groups, and the same trend was observed with the RNA-seq results.

### 3.5. GO and Pathway Enrichment Analyses of ASE Genes

To identify potential GO terms and pathways, a list of significant ASE genes (FDR < 0.05) was firstly submitted to DAVID (version 6.7) for biological processing and pathway enrichment. According to the ASE analysis results, 268 ASE genes related to MDV infection were finally obtained as initial input genes (Table S6). Using functional annotation clustering, 38 clusters were formed at the highest classification stringency. However, only seven clusters were chosen after using an enrichment cutoff > 1.0 (Table S7). GO terms and pathways analysis invoked in DAVID yielded 45 (5 terms of cellular component, 15 terms of molecular function, and 25 terms of biological processes) functional terms (*P* < 0.05, Table S8) and 11 pathways (*P* < 0.05, Table 8), including the immune-related pathway, T cell activation (Figure S1).

The ASE gene set was also submitted for IPA analysis. We obtained 215 pathways (*P* < 0.05, Table S9). The preeminent canonical pathways are comprised of T cell receptor (TCR), PI3K/AKT, B cell receptor (BCR), interleukin-4 (IL-4), and ERK/MAPK signaling pathways (Figures S2–S6). The corresponding *P*-values and ratios are listed in Table 9. The top diseases and disorder clusters, including inflammatory response, immunological disease, hematological disease, cancer, and organismal injury and abnormalities, were considered relevant to MDV infection. Additional information about the top networks, top lists, and top molecules were also obtained from the IPA analysis (Table S10).

## 4. Discussion

MD, a complicated tumor disease, has been used as a model to study human tumors [31]. The genetic mechanism underlying MD resistance and MD susceptibility is likely to be complex and remains incompletely understood. Thus, understanding the genetic basis of MD resistance or MD susceptibility for poultry is important to provide crucial clues for human diseases. In the present study, based on the high throughput sequencing platform, some bioinformatics analyses, such as ASE, DE, GO, and pathway analysis, were used to identify SNPs, genes, and enriched pathways from infected and non-infected birds. Six genes exhibiting ASE and six pathways were selected as the likely candidate factors that induce MD resistance. To minimize transcriptional variations and take full advantage of identical genetic backgrounds in inbred lines, we paired birds by reciprocal crossing and MDV infection and tested the differences. We not only identified genes most likely related to MD resistance, but also revealed the effects of MDV infection on the host.

ASE is a powerful technique used to measure the expression of each allele through studying SNP within an RNA sample [32,33,34,35]. The F_1_ data used here are an ideal test case for our approach. In this study, we hypothesized that a gene with allelic inequality or imbalance is the key genetic factor causing MDV susceptibility. Since variation in gene expression is thought to be a major factor affecting phenotypic variation, genes with ASE SNPs provide candidates and markers that may account for the complex trait of interest. Although the ASE SNPs identified here could not be considered to explain much more additive genetic variation of the genetic resistance to MD because fewer ASE SNPs and genes were obtained from RNA-seq data, our findings are still of interest in view of the strict experimental design and analysis methods. In this experiment, we successfully aligned RNA-seq reads to the reference chicken genome and identified high quality SNPs in infected and non-infected chickens. The significant SNPs exhibiting ASE in response to MDV infection were distributed on autosomes 1 to 28 and Z and W chromosomes of the chicken genome, with the number of SNPs for each chromosome roughly proportional to the size of each chromosome in the chicken genome assembly. To locate these SNPs on the chicken genome with respect to genes and classify them based on function, VEP was used. In the 6_3_ × 7_2_ I_N group, the most SNPs (*n* = 19, 31.1%) were classified as exonic; however, the largest number of SNPs (*n* = 52, 32.7%) was classified as intronic in the 7_2_ × 6_3_ I_N group. The largest number of SNPs was classified as intronic (*n* = 26, 34.7%) and located downstream of a gene (*n* =23, 20.5%) in infected and non-infected groups, respectively. Interestingly, for both ASE and DE results, more SNPs and DEGs were identified in the 7_2_ × 6_3_ I_N group than in the 6_3_ × 7_2_ I_N group, which may be because of the different MD incidences (%) of both reciprocal crosses. Seven overlapped ASE genes were identified in intercrosses 6_3_ × 7_2_ and 7_2_ × 6_3_ in response to MDV infection. We also identified 11 overlapped ASE genes in infected and non-infected groups, and these common genes are likely induced by MD viral infection in the host. *MCL1* was the only gene identified in both overlapped groups, which could be firstly selected as a key gene in the response to MDV infection. Other ASE genes, including *CD5*, *CD28*, *CD44*, *TCF1*, and *IRF-4*, are also important genes related to T cells, viruses, and tumors. DE is another powerful technique that could further explain the results of ASE. Many more overlapped genes were identified in DE than in ASE analysis in response to MDV infection, including *MCL1*.

We compared the results of the DEGs to the ASE gene analysis to identify four overlapped genes: *MCL1*, *SLC43A2*, *PDE3B*, and *ADAM33*. As a tumor disease, gene functional validation was conducted not only in CD4^+^ T cells but also in tumor tissues, which could provide powerful evidence of key genes responding to MDV resistance. Eight genes exhibiting ASE, including these four genes, showed significant differences in both CD4^+^ T cells and tumors of the comparison groups, and were completely consistent with the RNA-seq results. Therefore, these four genes could be selected as potential candidate genes to study MD resistance or MD susceptibility, of which *MCL1*, as a member of the *BCL2* family, plays a role in cell proliferation, differentiation, tumorigenesis, and apoptosis [36,37,38,39]. It is also associated with tumor, cancer, and CD4^+^ and CD8^+^ T cells [40,41,42,43]. In this study, we found an ASE SNP in this *MCL1* gene, and the expression of this gene is significantly higher in infected birds and tumors. Thus, this SNP may increase the gene expression of *MCL1* after MDV infection. This gene was identified both as a virus-related gene and a host-related gene, so it could be a critical gene connecting MDV to the host.

Chicken MHC plays an important role in the determination of resistance to MDV [1]. The GGA16 contains three loci, the B locus, Y (or Rfp-Y) locus, and nucleolar-organizing region (NOR), and only one ASE SNP was found to be associated with a gene that encodes for the class I alpha chain of the Rfp-Y loci [14]. In this study, no SNPs in the related loci were identified, and this could be attributed to the incomplete sequence information available for the GGA16 loci in the chicken genome. However, in the DE analysis, two MHC class II (MHCII)-associated genes, *BLB1* [44,45,46] and *DMB2* [47,48,49], were found to be significantly different between infected and non-infected birds, which are related to T cell immune responses, and their expressions are altered in cancers and tumors.

Due to the complexity of MD, it may not be a single-gene-controlled trait. Thus, we subsequently performed pathway analyses to investigate the specific gene sets involved in signaling cascades. DAVID analysis is usually a simple and useful method that is widely used for many gene set enrichment analyses. IPA is a powerful and useful commercial gene set enrichment analysis tool. In this study, based on the DAVID and IPA results, we focused on several important pathways that may be related to MDV infection. T cell activation [50] was identified by DAVID from the PANTHER pathway dataset. Activation of CD4^+^ T cells occurs through the simultaneous engagement of the T cell receptor and a co-stimulatory molecule on the T cell via the MHCII peptide and co-stimulatory molecules on the antigen-presenting cell (APC). This signaling pathway downstream from co-stimulatory molecules engages the PI3K/AKT molecules, which was identified in both DAVID and IPA analysis. The PI3K/AKT-mTOR signaling pathway is one of the three major signaling pathways critical in tumor progression. This classic route consists of two signaling pathways, PI3K/AKT and mTOR, which are important in regulating the cell cycle and directly related to cellular proliferation, tumors, and cancer. AKT is activated downstream of PI3K and then activates mTOR [51]. In many cancers, this pathway is overactive, thus reducing apoptosis and allowing proliferation. This pathway is related to the T cell activation pathway mentioned above and the key MD-related gene *MCL1*, identified in this study, is also involved in this pathway. This pathway may play a role in MD resistance or MD susceptibility. The TCR signaling pathway was the top canonical pathway identified by IPA. TCR is a molecule found on the surfaces of T cells or T lymphocytes, and is responsible for recognizing fragments of antigens as peptides bound to MHC molecules. When the TCR engages with an antigenic peptide and MHC (peptide/MHC), the T lymphocyte is activated through signal transduction, which is a series of biochemical events mediated by associated enzymes, co-receptors (like CD4), specialized adaptor molecules, and activated or released transcription factors [52]. MDV first uses B cells as targets for its replication before targeting activated CD4^+^ T cells. Thus, the BCR signaling pathway is critical for MDV infection. BCR is a transmembrane receptor protein located on the outer surface of B cells that has two crucial functions: signal transduction, involving changes in receptor oligomerization, and mediating internalization for subsequent processing of the antigen and presentation of peptides to helper T cells. BCR functions are required for normal antibody production, so defects in BCR signal transduction may lead to immunodeficiency [53]. The ERK/MAPK signaling pathway is a chain of proteins in the cell that communicates a signal from a receptor on the surface of the cell to the DNA in the nucleus of the cell. It consists of many proteins, including mitogen-activated protein kinase (MAPK), which is necessary for the development of cancers [54]. This pathway is one of the most extensively studied pathways involved in tumorigenesis [55] and has been found to be critical in previous MD studies by Yan et al. [56] and Subramaniam et al. [57,58]. Overall, our analyses indicate that the genes and pathways described here are worthy of further studies on MD.

## 5. Conclusions

In summary, we successfully identified SNPs associated with ASE and revealed genes and pathways that may be involved in genetic resistance to MD. Combining ASE, DE, and pathway analyses, and due to the complexity of MD, we ultimately found with high confidence that candidate genes (including *MCL1*, *SLC43A2*, *PDE3B*, *ADAM33*, *BLB1*, and *DMB2*, and especially *MCL1*) and several immune- or disease-related pathways (such as T cell activation, TCR, BCR, ERK/MAPK, and PI3K/AKT-mTOR signaling pathways) play potentially important roles in MDV infection. Overall, our approach underlines the importance of comprehensive functional studies in gaining valuable biological insight into the genetic factors behind MD and other complex traits. Our findings provide additional insights into the mechanisms of MD and disease resistance breeding in poultry.

## Figures and Tables

**Table 1:** Reads alignment.

Sample	No. of Total Reads	One Time Aligned Reads	Multiple Times Aligned Reads	Total Aligned Reads	Alignment
6_3_ × 7_2_ I_1	19,223,914	14,084,204	1,063,547	15,147,751	78.80%
6_3_ × 7_2_ I_2	18,840,597	13,938,069	1,061,498	14,999,567	79.61%
6_3_ × 7_2_ N_1	21,702,185	15,052,834	1,315,212	16,368,046	75.42%
6_3_ × 7_2_ N_2	28,813,734	18,928,310	2,143,823	21,072,133	73.13%
7_2_ × 6_3_ I_1	24,046,040	17,200,244	1,406,524	18,606,768	77.38%
7_2_ × 6_3_ I_2	31,386,432	22,780,475	1,935,946	24,716,421	78.75%
7_2_ × 6_3_ N_1	34,382,991	23,676,165	2,111,990	25,788,155	75.00%
7_2_ × 6_3_ N_2	25,854,955	17,753,865	1,747,443	19,501,308	75.43%

**Table 2:** Number of raw, screened, and heterozygous single nucleotide polymorphisms (SNPs).

Sample	Raw SNPs	No. of SNPs after Filtering	Heterozygous SNPs
6_3_ × 7_2_ I_1	552,541	165,244	65,689
6_3_ × 7_2_ I_2	541,775	162,552	62,759
6_3_ × 7_2_ N_1	507,400	156,293	64,405
6_3_ × 7_2_ N_2	577,350	184,980	75,044
7_2_ × 6_3_ I_1	614,547	199,101	81,324
7_2_ × 6_3_ I_2	716,655	259,509	105,297
7_2_ × 6_3_ N_1	671,763	250,528	100,882
7_2_ × 6_3_ N_2	561,954	184,962	73,707

**Table 3:** Number of ASE SNPs.

Group	SNPs for Chi-Square Test	No. of ASE SNPs (False Discovery Rate (FDR) < 0.05)	No. of ASE Genes
6_3_ × 7_2_ I vs 6_3_ × 7_2_ N	15,105	61	39
7_2_ × 6_3_ I vs 7_2_ × 6_3_ N	22,067	123	132
6_3_ × 7_2_ I vs 7_2_ × 6_3_ I	18,229	62	66
6_3_ × 7_2_ N vs 7_2_ × 6_3_ N	20,310	79	96

**Table 4:** Classification of ASE SNPs responding to Marek’s disease virus (MDV) infection.

SNP Type	6_3_ × 7_2_ I_N		7_2_ × 6_3_ I_N	
	SNPs	Genes	SNPs	Genes
Exonic	19	17	29	24
Intronic	16	13	52	43
Intergenic	17	-	4	-
Upstream	0	0	15	14
Downstream	0	0	36	32
5’ UTR	1	1	1	1
3’ UTR	6	6	20	16
Splicing	2	2	2	2
Total	61	39	159	132

UTR: Untranslated region.

**Table 5:** Classification of ASE SNPs between two reciprocal crosses.

SNP Type	Infected		Non-Infected	
	SNPs	Genes	SNPs	Genes
Exonic	12	11	20	18
Intronic	26	21	21	16
Intergenic	0	-	5	-
Upstream	11	10	21	18
Downstream	19	18	23	23
5’ UTR	0	0	0	0
3’ UTR	6	5	18	17
Splicing	1	1	4	4
Total	75	66	112	96

**Table 6:** Differentially expressed genes (DEGs) of four comparison groups.

Group	No. of DEGs (FDR < 0.05)	Up-/Down-Regulated
6_3_ × 7_2_ I vs 6_3_ × 7_2_ N	534	382/152
7_2_ × 6_3_ I vs 7_2_ × 6_3_ N	1543	854/689
Overlapped	223	153/70
6_3_ × 7_2_ I vs 7_2_ × 6_3_ I	328	279/49
6_3_ × 7_2_ N vs 7_2_ × 6_3_ N	20	5/15
Overlapped	0	0/0

**Table 7:** ASE and DE genes of four comparison groups.

Group	No. of ASE Genes	No. of DE Genes	Overlapped Genes
6_3_ × 7_2_ I vs 6_3_ × 7_2_ N	39	534	3
7_2_ × 6_3_ I vs 7_2_ × 6_3_ N	132	1543	24
6_3_ × 7_2_ I vs 7_2_ × 6_3_ I	66	328	10
6_3_ × 7_2_ N vs 7_2_ × 6_3_ N	96	20	0

**Table 8:** DAVID analysis of ASE genes in response to MDV infection (*P* < 0.05).

Category	Term	Count	%	*P*-Value
KEGG_PATHWAY	gga04914:Progesterone-mediated oocyte maturation	6	2.43	1.61E-02
KEGG_PATHWAY	gga04210:Apoptosis	6	2.43	1.71E-02
KEGG_PATHWAY	gga04810:Regulation of actin cytoskeleton	9	3.64	2.58E-02
KEGG_PATHWAY	gga04672:Intestinal immune network for IgA production	4	1.62	2.83E-02
KEGG_PATHWAY	gga04910:Insulin signaling pathway	7	2.83	2.86E-02
KEGG_PATHWAY	gga04114:Oocyte meiosis	6	2.43	4.37E-02
KEGG_PATHWAY	gga04070:Phosphatidylinositol signaling system	5	2.02	4.39E-02
PANTHER_PATHWAY	P00021:FGF signaling pathway	7	2.83	2.23E-03
PANTHER_PATHWAY	P00018:EGF receptor signaling pathway	6	2.43	8.78E-03
PANTHER_PATHWAY	P00048:PI3 kinase pathway	5	2.02	1.89E-02
PANTHER_PATHWAY	P00053:T cell activation	5	2.02	2.02E-02

**Table 9:** Top canonical pathways from IPA.

Ingenuity Canonical Pathways	*P*-value	Ratio
T Cell Receptor Signaling	1.00E-11	14/109 (12.8%)
PI3K/AKT Signaling	7.40E-10	13/124 (10.5%)
B Cell Receptor Signaling	1.34E-09	15/185 (8.1%)
IL-4 Signaling	2.65E-09	11/89 (12.4%)
ERK/MAPK Signaling	3.67E-09	15/199 (7.5%)

## Supplementary Materials

The following are available online at https://www.mdpi.com/2073-4425/10/9/718/s1. Figure S1. T cell activation pathway. Figure S2. T cell receptor signaling pathway. Genes in red are the significant genes identified in this study. Figure S3. PI3K/AKT signaling pathway. Genes in red are the significant genes identified in this study. Figure S4. mTOR signaling pathway. Genes in red are the significant genes identified in this study. Figure S5. B cell receptor signaling pathway. Genes in red are the significant genes identified in this study. Figure S6. ERK/MAPK signaling pathway. Genes in red are the significant genes identified in this study. Table S1. Gene-specific primers of the genes for the validation of RNA-seq results. Table S2. Gene-specific primers of the 11 overlapped genes for gene functional validation. Table S3. The detailed lists of ASE SNPs and genes. Table S4. The detailed list of DEGs. Table S5. The detailed list of overlapped genes between ASE and DE analyses. Table S6. The list of ASE initial input genes for GO and pathway analyses. Table S7. The detailed list of clusters with enrichment scores >1.0 from DAVID. Table S8. The detailed list of GO terms (*P* < 0.05) from DAVID. Table S9. The detailed list of Ingenuity Canonical Pathways (*P* < 0.05) from the IPA analysis. Table S10. The detailed information of the top canonical pathways, top upstream regulators, top diseases, top networks, and top tox lists from the IPA analysis.

## References

[1] Bacon L.D., Hunt H.D., Cheng H.H. (2000). A Review of the Development of Chicken Lines to Resolve Genes Determining Resistance to Diseases. Poult. Sci..

[2] Calnek B.W., Harai K. (2001). Pathogenesis of Marek’s Disease Virus Infection. Marek’s Disease.

[3] Calnek W.B., Witter R.L. (1985). Marek’s Disease—A Model for Herpesvirus Oncology. CRC Crit. Rev. Microbiol..

[4] Shek W.R., Calnek B.W., Schat K.A., Chen C.H. (1983). Characterization of Marek’s Disease Virus-Infected Lymphocytes: Discrimination between Cytolytically and Latently Infected Cells. J. Natl. Cancer Inst..

[5] Calnek B.W., Schat K.A., Ross L.J.N., Shek W.R., Chen C.L. (1984). Further Characterization of Marek’s Disease Virus-Infected Lymphocytes. I. In Vivo Infection. Int. J. Cancer.

[6] Calnek B.W., Schat K.A., Ross L.J.N., Chen C.L. (1984). Further Characterization of Marek’s Disease Virus-Infected Lymphocytes. Ii. In Vitro Infection. Int. J. Cancer.

[7] Schat K.A., Chen C.L., Calnek B.W., Char D. (1991). Transformation of T-Lymphocyte Subsets by Marek’s Disease Herpesvirus. J. Virol..

[8] Main B.J., Bickel R.D., McIntyre L.M., Graze R.M., Calabrese P.P., Nuzhdin S.V. (2009). Allele-Specific Expression Assays Using Solexa. BMC Genom..

[9] Bell C.G., Beck S. (2009). Advances in the Identification and Analysis of Allele-Specific Expression. Genome Med..

[10] Bjornsson H.T., Albert T.J., Ladd-Acosta C.M., Green R.D., Rongione M.A., Middle C.M., Irizarry R.A., Broman K.W., Feinberg A.P. (2008). SNP-Specific Array-Based Allele-Specific Expression Analysis. Genome Res..

[11] Heap G.A., Yang J.H.M., Downes K., Healy B.C., Hunt K.A., Bockett N., Franke L., Dubois P.C., Mein C.A., Dobson R.J. (2010). Genome-Wide Analysis of Allelic Expression Imbalance in Human Primary Cells by High-Throughput Transcriptome Resequencing. Hum. Mol. Genet..

[12] Cheng H.H., Perumbakkam S., Pyrkosz A.B., Dunn J.R., Legarra A., Muir W.M. (2015). Fine Mapping of Qtl and Genomic Prediction Using Allele-Specific Expression Snps Demonstrates That the Complex Trait of Genetic Resistance to Marek’s Disease Is Predominantly Determined by Transcriptional Regulation. BMC Genom..

[13] MacEachern S., Muir W.M., Crosby S.D., Cheng H.H. (2012). Genome-Wide Identification and Quantification of Cis-and Trans-Regulated Genes Responding to Marek’s Disease Virus Infection Via Analysis of Allele-Specific Expression. Front. Genet..

[14] Perumbakkam S., Muir W.M., Black-Pyrkosz A., Okimoto R., Cheng H.H. (2013). Comparison and Contrast of Genes and Biological Pathways Responding to Marek’s Disease Virus Infection Using Allele-Specific Expression and Differential Expression in Broiler and Layer Chickens. BMC Genom..

[15] Stone H.A. (1975). Use of Highly Inbred Chickens in Research: Agricultural Research Service.

[16] Witter R.L., Calnek B.W., Buscaglia C., Gimeno I.M., Schat K.A. (2005). Classification of Marek’s Disease Viruses According to Pathotype: Philosophy and Methodology. Avian Pathol..

[17] Meyer L.R., Zweig A.S., Hinrichs A.S., Karolchik D., Kuhn R.M., Wong M., Sloan C.A., Rosenbloom K.R., Roe G., Rhead B. (2013). The UCSC Genome Browser Database: Extensions and Updates 2013. Nucleic Acids Res..

[18] Schmieder R., Edwards R. (2011). Quality Control and Preprocessing of Metagenomic Datasets. Bioinformatics.

[19] Gordon A., Hannon G.J., Fastx-Toolkit FASTQ/A Short-Reads Preprocessing Tools. http://hannonlab.cshl.edu/fastx_toolkit2010.

[20] Bolger A.M., Lohse M., Usadel B. (2014). Trimmomatic: A Flexible Trimmer for Illumina Sequence Data. Bioinformatics.

[21] Langmead B., Salzberg S.L. (2012). Fast Gapped-Read Alignment with Bowtie 2. Nat. Methods.

[22] Langdon W.B. (2015). Performance of Genetic Programming Optimised Bowtie2 on Genome Comparison and Analytic Testing (Gcat) Benchmarks. BioData Min..

[23] Li H., Handsaker B., Wysoker A., Fennell T., Ruan J., Homer N., Marth G., Abecasis G., Durbin R. (2009). The Sequence Alignment/Map Format and Samtools. Bioinformatics.

[24] Simsek U.G., Ciftci M., Cerci I.H., Bayraktar M., Dalkilic B., Arslan O., Balci T.A. (2011). Impact of Stocking Density and Feeding Regimen on Broilers: Performance, Carcass Traits and Bone Mineralisation. J. Appl. Anim. Res..

[25] McKenna A., Hanna M., Banks E., Sivachenko A., Cibulskis K., Kernytsky A., Garimella K., Altshuler D., Gabriel S., Daly M. (2010). The Genome Analysis Toolkit: A Mapreduce Framework for Analyzing Next-Generation DNA Sequencing Data. Genome Res..

[26] Danecek P., Auton A., Abecasis G., Albers C.A., Banks E., DePristo M.A., Handsaker R.E., Lunter G., Marth G.T., Sherry S.T. (2011). The Variant Call Format and Vcftools. Bioinformatics.

[27] McLaren W., Pritchard B., Rios D., Chen Y., Flicek P., Cunningham F. (2010). Deriving the Consequences of Genomic Variants with the Ensembl API and SNP Effect Predictor. Bioinformatics.

[28] Anders S., Pyl P.T., Huber W. (2014). HTSeq-a Python Framework to Work with High-Throughput Sequencing Data. Bioinformatics.

[29] Robinson M.D., McCarthy D.J., Smyth G.K. (2010). edgeR: A Bioconductor Package for Differential Expression Analysis of Digital Gene Expression Data. Bioinformatics.

[30] Huang D.W., Sherman B.T., Lempicki R.A. (2009). Systematic Integrative Analysis of Large Gene Lists Using David Bioinformatics Resources. Nat. Protoc..

[31] Osterrieder N., Kamil J.P., Schumacher D., Tischer B.K., Trapp S. (2006). Marek’s Disease Virus: From Miasma to Model. Nat. Rev. Microbiol..

[32] Hasin-Brumshtein Y., Hormozdiari F., Martin L., van Nas A., Eskin E., Lusis A.J., Drake T.A. (2014). Allele-Specific Expression and Eqtl Analysis in Mouse Adipose Tissue. BMC Genom..

[33] Serre D., Gurd S., Ge B., Sladek R., Sinnett D., Harmsen E., Bibikova M., Chudin E., Barker D.L., Dickinson T. (2008). Differential Allelic Expression in the Human Genome: A Robust Approach to Identify Genetic and Epigenetic Cis-Acting Mechanisms Regulating Gene Expression. PLoS Genet..

[34] Pant P.K., Tao H., Beilharz E.J., Ballinger D.G., Cox D.R., Frazer K.A. (2006). Analysis of Allelic Differential Expression in Human White Blood Cells. Genome Res..

[35] Degner J.F., Marioni J.C., Pai A.A., Pickrell J.K., Nkadori E., Gilad Y., Pritchard J.K. (2009). Effect of Read-Mapping Biases on Detecting Allele-Specific Expression from Rna-Sequencing Data. Bioinformatics.

[36] Craig R.W. (2002). MCL1 Provides a Window on the Role of the Bcl2 Family in Cell Proliferation, Differentiation and Tumorigenesis. Leukemia.

[37] Kozopas K.M., Yang T., Buchan H.L., Zhou P., Craig R.W. (1993). MCL1, a Gene Expressed in Programmed Myeloid Cell Differentiation, Has Sequence Similarity to BCL2. Proc. Natl. Acad. Sci. USA.

[38] Lee R., Gillet G., Burnside J., Thomas S.J., Neiman P. (1999). Role of Nr13 in Regulation of Programmed Cell Death in the Bursa of Fabricius. Genes Dev..

[39] Zhong Y., Liao Y., Fang S., Tam J.P., Liu D. (2012). Up-Regulation of MCL1 and Bak by Coronavirus Infection of Human, Avian and Animal Cells Modulates Apoptosis and Viral Replication. PLoS ONE.

[40] Ertel F., Nguyen M., Roulston A., Shore G.C. (2013). Programming Cancer Cells for High Expression Levels of MCL1. EMBO Rep..

[41] Krajewski S., Krajewska M., Ehrmann J., Sikorska M., Lach B., Chatten J., Reed J.C. (1997). Immunohistochemical Analysis of Bcl-2, Bcl-X, Mcl-1, and Bax in Tumors of Central and Peripheral Nervous System Origin. Am. J. Pathol..

[42] Rassidakis G.Z., Lai R., McDonnell T.J., Cabanillas F., Sarris A.H., Medeiros L.J. (2002). Overexpression of MCL1 in Anaplastic Large Cell Lymphoma Cell Lines and Tumors. Am. J. Pathol..

[43] Tripathi P., Koss B., Opferman J.T., Hildeman D.A. (2013). MCL1 Antagonizes Bax/Bak to Promote Effector CD4^+^ and CD8^+^ T-Cell Responses. Cell Death Differ..

[44] Zhou H., Lamont S.J. (2003). Chicken Mhc Class I and Ii Gene Effects on Antibody Response Kinetics in Adult Chickens. Immunogenetics.

[45] Xu R., Li K., Chen G., Xu H., Qiang B., Li C., Liu B. (2007). Characterization of Genetic Polymorphism of Novel MHC B-LB II Alleles in Chinese Indigenous Chickens. J. Genet. Genom..

[46] Jacob J.P., Milne S., Beck S., Kaufman J. (2000). The Major and a Minor Class II β-Chain (B-LB) Gene Flank the Tapasin Gene in the BF/BL Region of the Chicken Major Histocompatibility Complex. Immunogenetics.

[47] Pezeshki A.M., Côté M.H., Azar G.A., Routy J.P., Boulassel M.R., Thibodeau J. (2011). Forced Expression of Hla-Dm at the Surface of Dendritic Cells Increases Loading of Synthetic Peptides on MHC Class II Molecules and Modulates T Cell Responses. J. Immunol..

[48] Amria S., Hajiaghamohseni L.M., Harbeson C., Zhao D., Goldstein O., Blum J.S., Haque A. (2008). HLA-DM Negatively Regulates HLA-DR4-restricted Collagen Pathogenic Peptide Presentation and T Cell Recognition. Eur. J. Immunol..

[49] Chazara O., Tixier-Boichard M., Morin V., Zoorob R., Bed’Hom B. (2011). Organisation and Diversity of the Class II DM Region of the Chicken MHC. Mol. Immunol..

[50] Smith-Garvin J.E., Koretzky G.A., Jordan M.S. (2009). T Cell Activation. Annu. Rev. Immunol..

[51] Rafalski V.A., Brunet A. (2011). Energy Metabolism in Adult Neural Stem Cell Fate. Prog. Neurobiol..

[52] Kindt T.J., Goldsby R.A., Osborne B.A., Kuby J. (2007). Kuby Immunology.

[53] Seda V., Mraz M. (2015). B-Cell Receptor Signalling and Its Crosstalk with Other Pathways in Normal and Malignant Cells. Eur. J. Haematol..

[54] Orton R.J., Sturm O.E., Vyshemirsky V., Calder M., Gilbert D.R., Kolch W. (2005). Computational Modelling of the Receptor-Tyrosine-Kinase-Activated Mapk Pathway. Biochem. J..

[55] Dhillon A.S., Hagan S., Rath O., Kolch W. (2007). Map Kinase Signalling Pathways in Cancer. Oncogene.

[56] Yan Y., Yang N., Cheng H.H., Song J., Qu L. (2015). Genome-Wide Identification of Copy Number Variations between Two Chicken Lines That Differ in Genetic Resistance to Marek’s Disease. BMC Genom..

[57] Subramaniam S., Johnston J., Preeyanon L., Brown C.T., Kung H.-J., Cheng H.H. (2013). Integrated Analyses of Genome-Wide DNA Occupancy and Expression Profiling Identify Key Genes and Pathways Involved in Cellular Transformation by a Marek’s Disease Virus Oncoprotein, Meq. J. Virol..

[58] Subramaniam S., Preeyanon L., Cheng H.H. (2013). Transcriptional Profiling of Meq-Dependent Genes in Marek’s Disease Resistant and Susceptible Inbred Chicken Lines. PLoS ONE.

