# Supplementary figures and images for: Allele-Specific Expression of CD4+ T Cells in Response to Marek’s Disease Virus Infection
Source: Genes (Basel). 2019 Sep 17;10(9):718. doi: 10.3390/genes10090718 (PMC6770979; doi:10.3390/genes10090718)

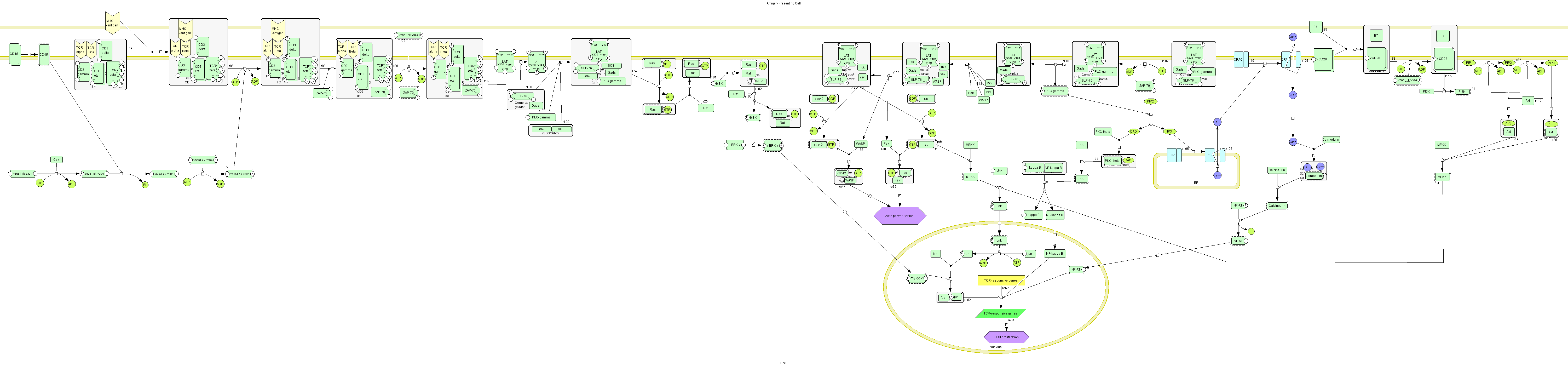

Supplement: Supplementary file 1 [file genes-10-00718-s001.zip › Supplementary Files/Figure S1.tif]

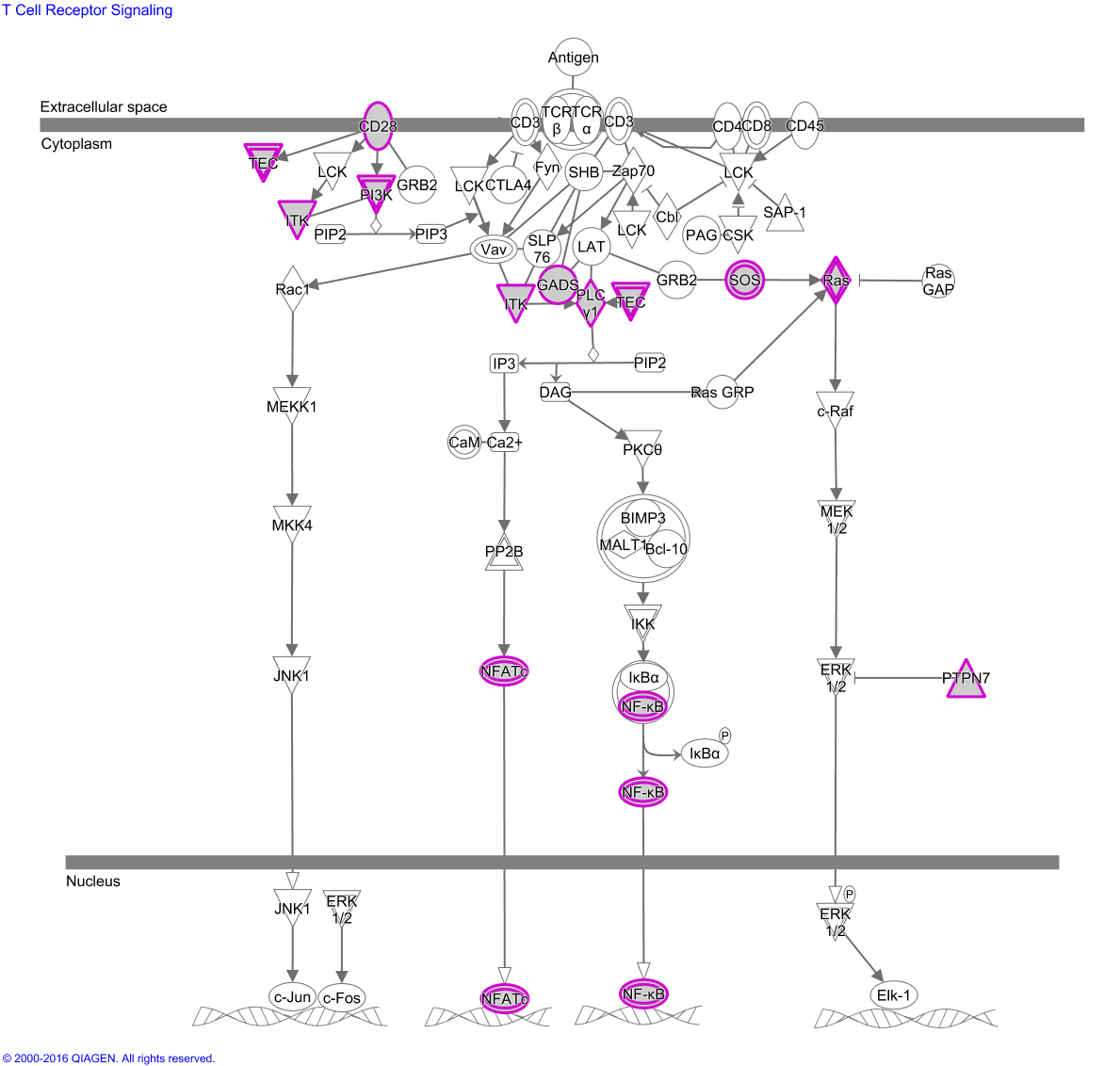

Supplement: Supplementary file 1 [file genes-10-00718-s001.zip › Supplementary Files/Figure S2.tif]

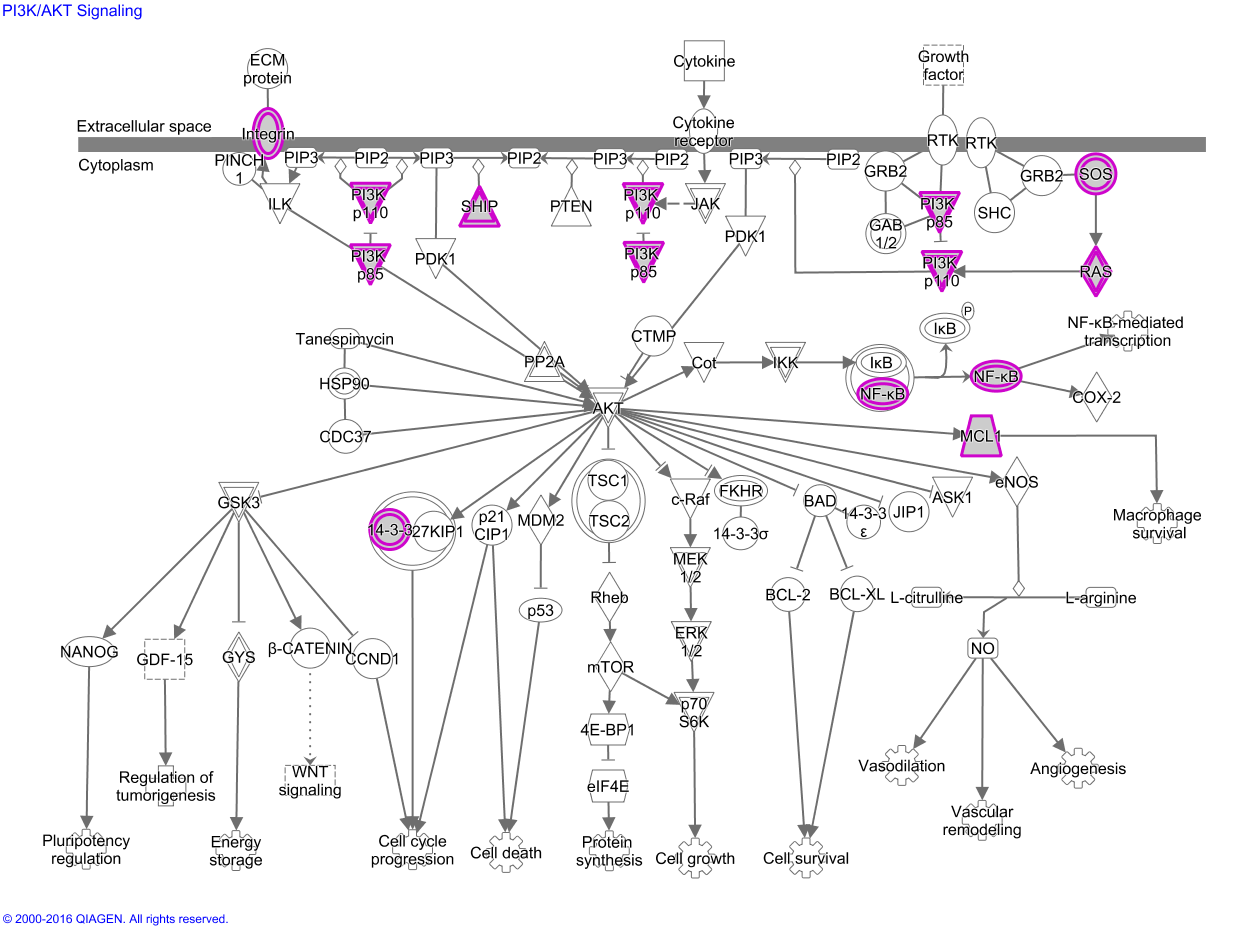

Supplement: Supplementary file 1 [file genes-10-00718-s001.zip › Supplementary Files/Figure S3.tif]

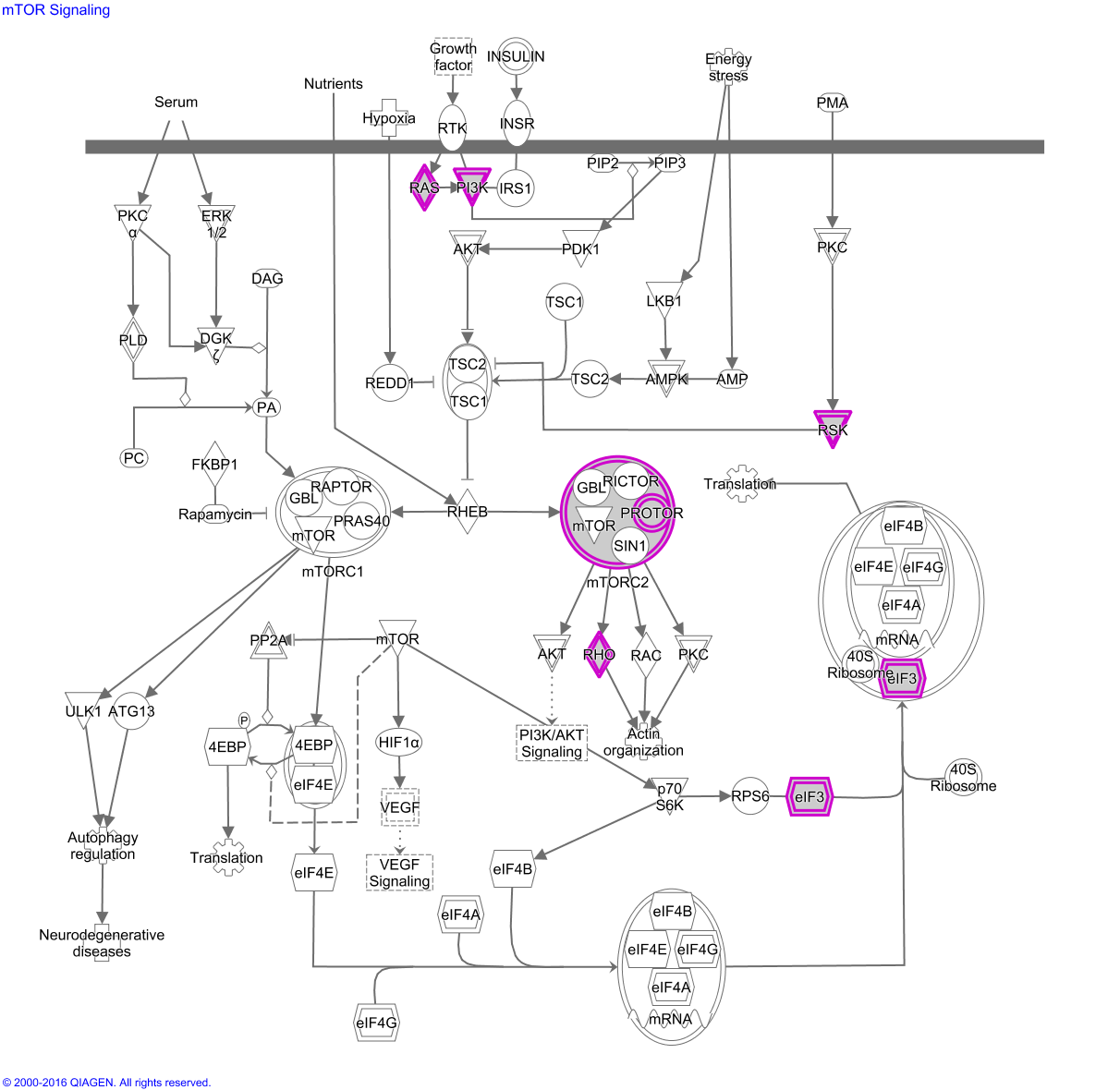

Supplement: Supplementary file 1 [file genes-10-00718-s001.zip › Supplementary Files/Figure S4.tif]

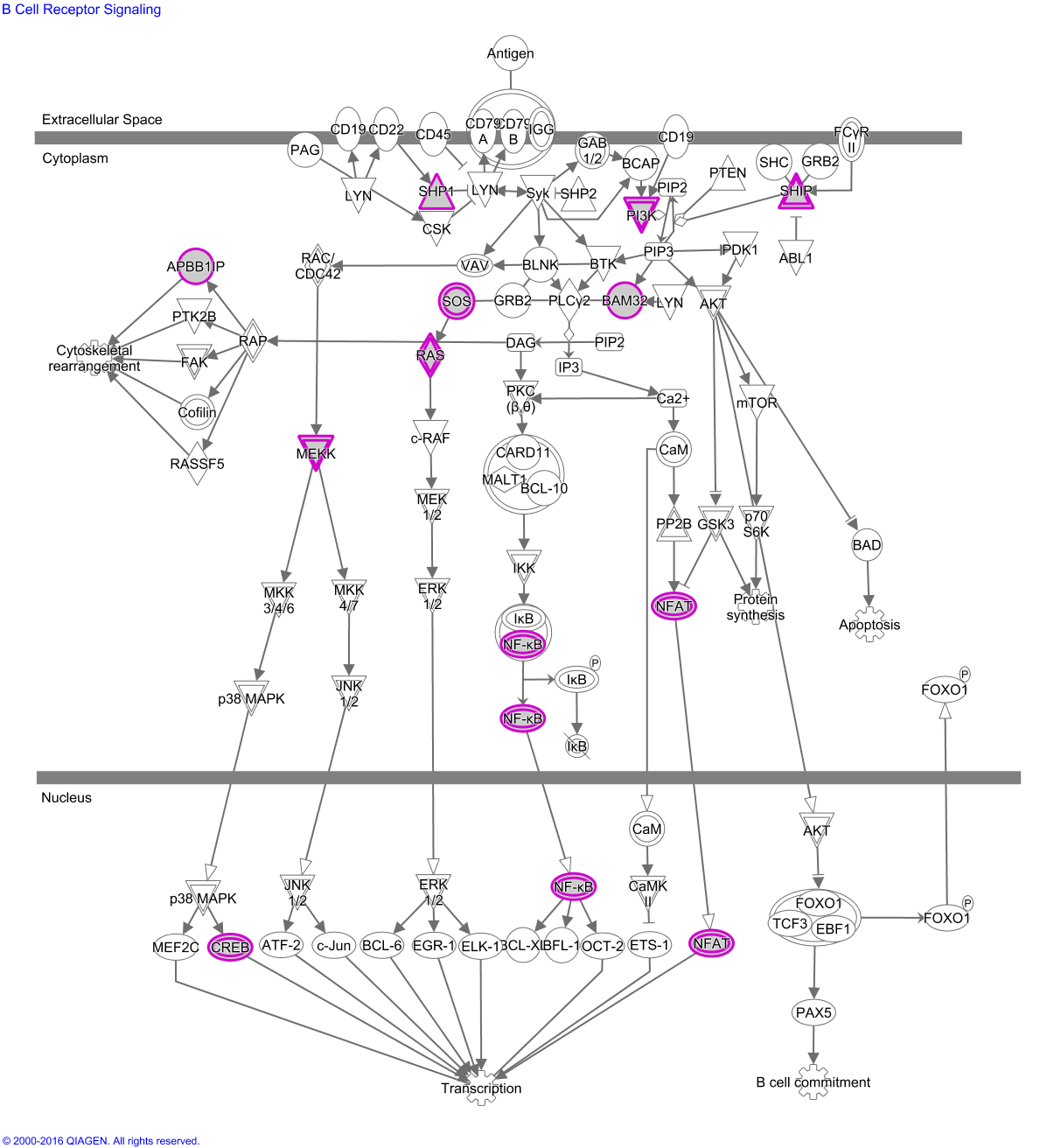

Supplement: Supplementary file 1 [file genes-10-00718-s001.zip › Supplementary Files/Figure S5.tif]

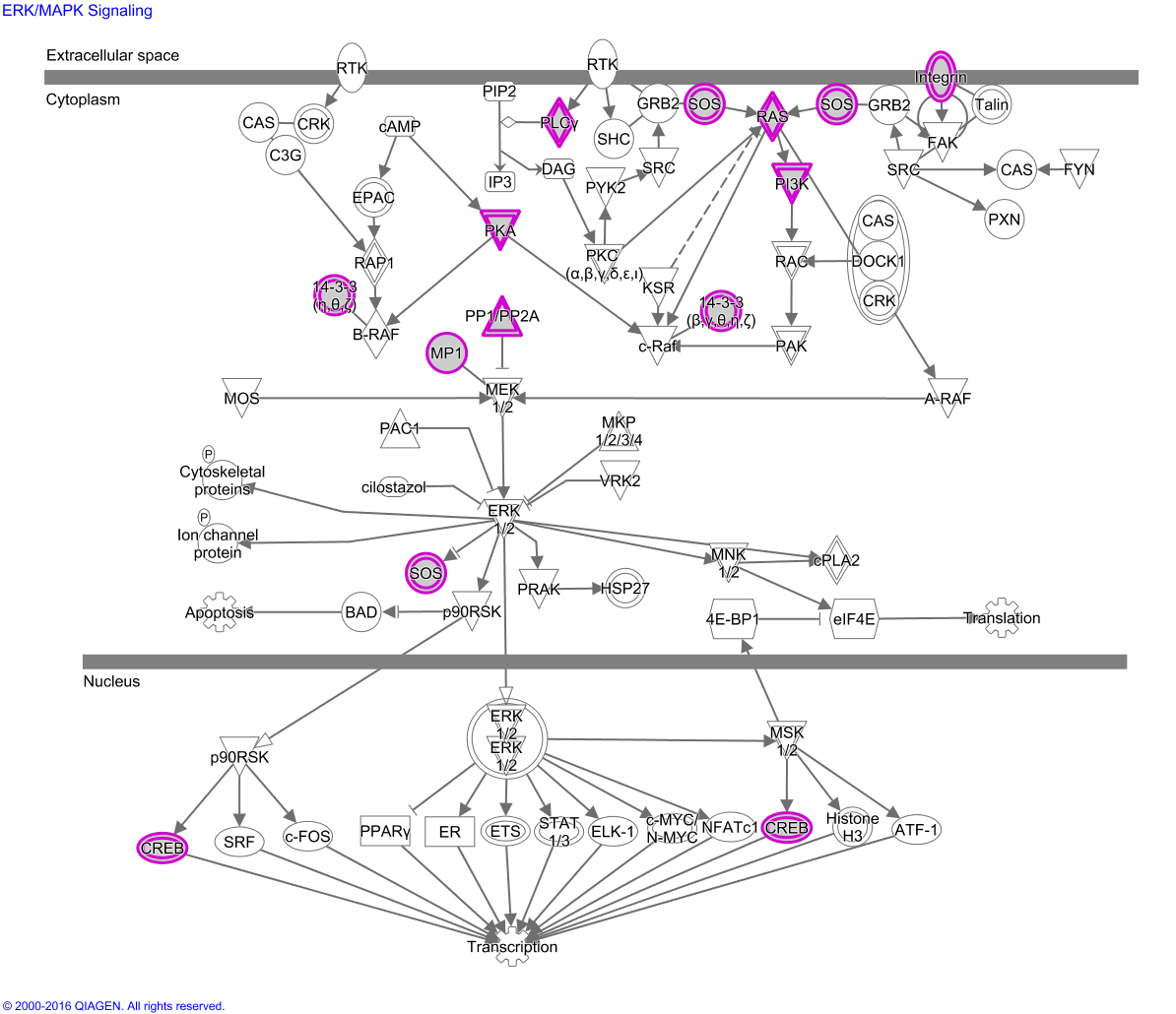

Supplement: Supplementary file 1 [file genes-10-00718-s001.zip › Supplementary Files/Figure S6.tif]
